# Molecular Regulation of Differentiation in Early B-Lymphocyte Development

**DOI:** 10.3390/ijms19071928

**Published:** 2018-06-30

**Authors:** Mikael Sigvardsson

**Affiliations:** 1 Division of Molecular Hematology, Lund Stem Cell Center, Department of Laboratory Medicine, Lund University, 22184 Lund, Sweden; mikael.sigvardsson@med.lu.se; Tel.: +46-708-320-120; 2 Department of Clinical and Experimental Medicine, Linköping University, SE-581 85 Linköping, Sweden

**Keywords:** B-lymphocyte, development, transcription factors, lymphoid leukemia

## Abstract

B-lymphocyte differentiation is one of the best understood developmental pathways in the hematopoietic system. Our understanding of the developmental trajectories linking the multipotent hematopoietic stem cell to the mature functional B-lymphocyte is extensive as a result of efforts to identify and prospectively isolate progenitors at defined maturation stages. The identification of defined progenitor compartments has been instrumental for the resolution of the molecular features that defines given developmental stages as well as for our understanding of the mechanisms that drive the progressive maturation process. Over the last years it has become increasingly clear that the regulatory networks that control normal B-cell differentiation are targeted by mutations in human B-lineage malignancies. This generates a most interesting link between development and disease that can be explored to improve diagnosis and treatment protocols in lymphoid malignancies. The aim of this review is to provide an overview of our current understanding of molecular regulation in normal and malignant B-cell development.

## 1. Introduction

The generation of B-lymphocytes in the bone marrow (BM) is a highly complex process guiding multipotent hematopoietic stem cells to become immunoglobulin-expressing B-cells. The differentiation process depends on the orchestrated activities of transcription factor (TF) networks and extracellular signals acting in conjunction to drive the expansion and maturation of progenitor populations. The process is complicated by the fact that cells must undergo *Immunoglobulin* (Ig) gene recombination and both positive and negative selection events to ensure proper functionality (reviewed in [1]). Even though much of our understanding of this developmental pathway is based on mouse models, there exist several similarities between mouse and human B-cell differentiation [2,3,4]. Furthermore, it is now evident that the same mechanisms that control normal B-lymphoid development in mice and humans are targeted in B-lymphoid malignancies (reviewed in [5]). The aim of this review is to provide an overview of our knowledge about developmental trajectories and regulatory networks in normal early B-lymphocyte development and their potential involvement in malignant transformation.

## 2. Resolving Developmental Trajectories in B-Cell Development

In order to understand the process controlling the generation of highly specified blood cells, it is of critical importance to identify and prospectively isolate cells at defined maturation stages. B-lymphocyte development has been suggested to proceed from the hematopoietic stem cell, through the lymphoid primed multipotent progenitor (LMPP) [6] stage, to generate a lymphoid-restricted common lymphoid progenitor (CLP) [7]. CLPs have the capacity to generate B-lineage-restricted B220^+^ Fraction A compartment [8], proceeding in differentiation to generate CD19^+^ cells.

While the progenitor cells within the classical CLP compartment retain lymphoid linage potentials and display a reduced capacity to generate myeloid cells [7], the inclusion of additional surface markers in the staining protocols has revealed a molecular and functional heterogeneity within this population. Surface expression of Integrin α(2)β(7) (LPAM1) or CXCR6 identifies a subpopulation of cells with reduced B but preserved NK/T lineage potential [9], and BST2 expression identifies a dendritic cell population [10]. It is further possible to isolate a B220^+^ population with preserved combined B and T-lineage potential within the classical CLP compartment [11,12]. Hence, it has become increasingly clear that the CLP compartment is highly heterogeneous and likely harbors a variety of more or less lineage-restricted progenitors.

**Figure 1:**
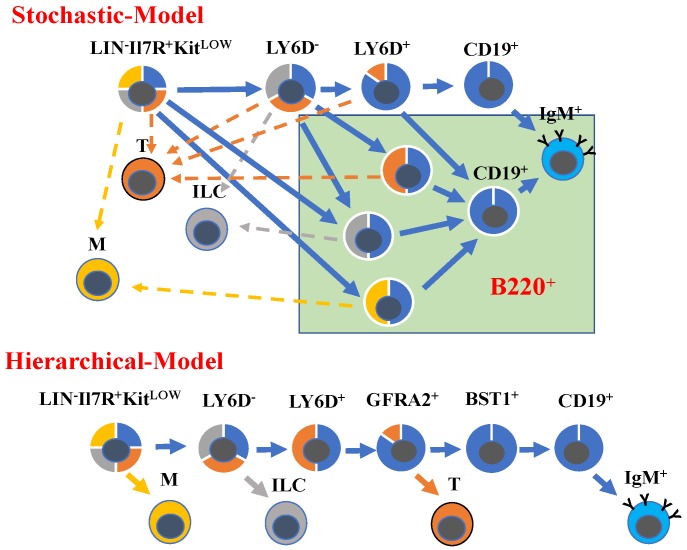
Developmental trajectories in B-cell development. Schematic drawing displaying two models for the developmental trajectories in B-cell development. Yellow indicates myeloid potential (M), gray indicates potential to generate innate lymphoid cells (ILC), orange indicates T lineage potential (T), and blue indicates B-cell potential. The arrows indicate potential developmental trajectories for the defined lineages. The green square indicates B220^+^ populations.

One of the earliest markers associated with B-cell progenitors is B220, a heavily glycosylated splice form of the CD45 protein (CD45R) (reviewed in [13]). Expression of B220 in combination with other surface markers, such as CD43 (S7), CD24 (HSA), BP1, CD19, KIT (CD117), CD93 (AA4.1) [8,14,15,16], and CD25 [17,18], can be used to identify specific subpopulations of B-cell progenitors. Combined with functional and molecular analysis this has allowed for the establishment of a developmental hierarchy instrumental for our understanding of B-cell development (Figure 1). However, while a substantial fraction of the CD19^−^ B-cell progenitors express B220, functional analysis fails to link B220 expression exclusively to B-lineage-committed progenitors. Rather, a fraction of the B220^+^ cells retain T-cell [11,12,15], NK [19], and even myeloid potential [20,21]. 

These findings could be seen as evidence that early B-cell development does not follow one distinct path but rather proceeds through multiple pathways whereby lineage potentials are lost in a more or less stochastic manner (Figure 1). This model for lymphocyte development is supported by the finding that early thymic progenitors display combined T-macrophage potential but most have a limited ability to generate B-lineage cells [22]. Furthermore, the fetal liver contains cells with combined B-macrophage or T-macrophage potential [23]. Additional complexity in developmental trajectories in the fetal liver comes with the identification of B/T and B/NK bi-potent progenitors [9,24]. Hence, the difficulty of identifying CD19^−^ B-lineage committed progenitors could be a consequence of non-linear developmental paths not subject to the restrictions predicted from a hematopoietic tree (Figure 1). 

While conventional surface marker expression did not allow for the prospective isolation of committed CD19^−^ B-cell progenitors, expression of a reporter gene under the control of the Igll1 (Lambda 5) promoter [25] allowed for the identification of B-lymphoid- restricted progenitors within the classical CLP compartment [26]. Despite the fact that the *Igll1* gene, encoding one of the surrogate light chains [27], is not crucial for the earliest stages of B-cell development [28,29], the gene is transcribed in primitive progenitors serving as a marker for lineage commitment [30,31]. Continued analysis of the cellular heterogeneity within the CLP compartment identified the surface marker Ly6D as being expressed in a subpopulation of cells [32]. Transplantation experiments revealed that the Ly6D^+^ “CLP” population was largely restricted to the production of B-lineage cells; subsequently, these progenitors were denoted B-lymphocyte progenitors BLPs [32]. While the BLP population displayed a minimal T-linage potential in vivo, in vitro differentiation analysis suggested that a fraction of the cells retained the ability to generate T-lineage cells in response to a strong Notch signal [12]. This indicates further heterogeneity within the BLP compartment. This heterogeneity is largely resolved using the expression of the surface markers GNDF family receptor α2 (GFRA2) and bone marrow stroma cell antigen 1 (BST1), to prospectively isolate BLP1, BLP2, and BLP3 cells displaying a progressive degree of commitment to B-cell development [33] (Figure 1). Even though the progenitor populations defined by GFRA2 and BST1 displayed variable levels of B220, their gene expression patterns as well as functional analysis suggest that they represent highly similar or even identical developmental stages. These findings would be in line with observations made using multiparameter molecular analysis of human B-lineage development suggesting a hierarchical model based on progressive and ordered loss of lineage potentials (Figure 1) [34].

While CD19 expression marks stably committed B-lineage progenitors, this population is complex and can be further subdivided into defined developmental stages. The most immature cells express KIT, CD127 (IL7Ra), CD49E, CD11A, CD54, and CD43 [17,18,35,36], while CD25 (IL2Rα) is restricted to cells with a functional pre-BCR [17]. The expression of CD2 resembles that of CD25 because it is restricted to cells with cytoplasmic immunoglobulin heavy (IgH) chains [37]. Despite that surface antigens are most useful in the identification of progenitor stages in early B-cell development, a degree of heterogeneity can be observed even within a defined developmental stage [18]. This argues for the use of multiple markers for the identification of any given progenitor population. In all, the development of protocols for the prospective isolation of defined progenitor compartments provides a detailed map of the developmental trajectories in B-cell development generally compatible with the hematopoietic tree.

## 3. Transcription Factor Networks Regulating Early B-Cell Development

**Figure 2:**
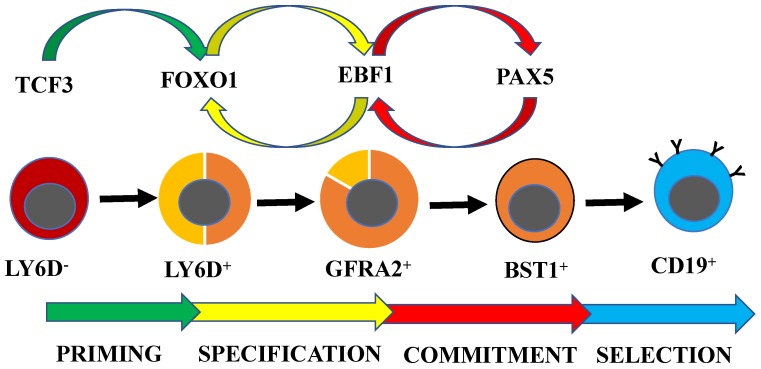
Schematic drawing of the transcription factor networks involved in priming (green arrows), specification (yellow arrows), commitment (red arrows), and selection (blue arrows) in early B-cell development. Red indicates B/T and NK cell potential, orange indicates B-cell potential, and yellow indicates residual T-cell potential. Blue indicates IgM^+^ cells.

With our increasing understanding of the developmental trajectories in early B-cell development, it has been possible to explore molecular interplay at defined developmental stages. While lineage-specific gene expression is often associated with functional lineage commitment, it has been reported that the expression of lineage-associated genes can be detected already in multipotent progenitors [38,39]. These early transcriptional programs are suggested to be associated with defined cell fates [40], indicating the existence of functional lineage priming in non-committed progenitors (Figure 2). Lymphoid lineage priming is mainly observed as activation of genes expressed in both B- and T-cells, including *Rag1* and *Dntt* [39,41]. The activation of these genes is dependent on the TFs TCF3 [42,43,44], IKZF1 (IKAROS) [45], SPI1 (PU.1) [46,47], and MYB [48] acting in a concerted manner to promote the development of lymphoid progenitors. While SPI1 and IKZF1 create a regulatory loop controlling lymphoid versus myeloid cell fate [49], TCF3 initiates the B-lineage-restricted transcriptional program by activation of the *FoxO1* gene [50] (Figure 2). FOXO1 acts in a feed-forward loop with the TF EBF1 [51] to activate the transcription of B-lineage genes during B-cell specification [52,53,54]. The ability of EBF1 to coordinate the activation of transcriptionally inactive genes in epigenetically silenced chromatin [55,56] is likely a consequence of EBF1 associating with chromatin remodeling complexes, thereby directly impacting the structure and the epigenetic landscape [57,58]. Despite the LY6D^+^ “CLP” compartment being intact or even increased in the absence of EBF1 [59,60], the GFRA2^+^ compartment is dramatically decreased [33], the transcription of B-lineage-restricted genes is lost [51,52], and the cells are not properly lineage-restricted [60]. This highlights the essential role of EBF1 in B-lineage specification.

Even though EBF1 has the ability to repress genes associated with alternative cell fates [61,62] and the loss of EBF1 in B-lineage cells results in plasticity [63], stable lineage commitment depends on the TF PAX5. Despite a large portion of the B-lineage-restricted transcriptional program being activated in the absence of PAX5 [64,65], the progenitor cells are not stably committed to B-lineage cell fate [65,66,67,68,69]. In vitro differentiation or transplantation of PAX5-deficient pro-B cells, as well as deletion of the *Pax5* gene in B-lineage cells, results in the formation of both myeloid and T-lineage cells in vivo and in vitro [65,66,67,68,69,70,71]. Lineage restriction is likely achieved through direct repression of target genes such as *Colony-stimulating factor receptor 1* (*Csf1r* or *c-fms*) gene [72] and *Notch1* [73]. Even though this suggests that lineage specification can be separated from commitment [65], the finding that PAX5 is a direct EBF1 target [74] links these two processes in normal development. PAX5 and EBF1 target control elements in the *Ebf1* gene [75,76], creating a second regulatory loop and resulting in functional lineage commitment (Figure 2). The importance of the reciprocal regulation and collaboration between EBF1 and PAX5 is highlighted by the finding that normal as well as malignant pro-B cells carrying trans heterozygote mutations in the *Ebf1* and *Pax5* genes display lineage plasticity [77,78].

In addition to their function in stable lineage commitment, EBF1 and PAX5 are critical regulators of genes encoding proteins forming the pre-B cell receptor (pre-BCR) [54,79,80,81,82]. This receptor is formed as a newly generated IGH chain complex with surrogate light chains IGLL1 (λ5) and VPREB as well as the signal transduction proteins CD79α and CD79β (reviewed in [1,83]). Combined signaling through the pre-BCR and the IL7 receptor stimulates a proliferative burst [84] and causes a reduction in RAG protein levels [85]. While the proliferative burst is of great importance for the expansion and overall production of B-lineage cells, progressive development and Ig light chain rearrangement depend on the cells exiting the cell cycle [86,87]. This maturation step is suggested to depend on a pre-BCR-mediated activation of a regulatory network involving interferon regulatory factor (IRF4) and PAX5 [88]. PAX5 targets and activates the *Ikzf3* gene [89], encoding a TF suggested to collaborate with IKZF1 to repress genes encoding surrogate light chain components [90]. This results in a reduction of pre-BCR levels on the surface of the pro-B cell, reducing the proliferative signal. IRF4 has a somewhat different role in this process since it collaborates with the transcription factor FOXO1, stabilized by the pre-BCR signal [88], to drive differentiation and reactivate *Rag* gene expression critical for recombination of the Ig light chain genes [91,92,93]. Furthermore, IRF4 increases the expression of the CXCL12-responsive chemokine receptor CXCR4 [87]. This has been suggested to stimulate migration of the pre-B cells to a micromolecular niche with low levels of Il7 to further reduce the proliferative signal [87]. Hence, B-cell development is driven by an intricate interplay between stage-specific regulatory TF networks that orchestrate the differentiation process.

## 4. Transcription Factor Networks Link Development to B-Lymphoid Malignancies

While these TF networks clearly play crucial roles in normal B-cell development, it is becoming increasingly clear that they are closely connected to malignant transformation (reviewed in [5]). This is because genetic alterations in the *PAX5*, *EBF1*, or *IKZF1* genes are observed in a majority of B-ALL patients [94,95,96]. Even though there are reports of translocations of TF coding genes resulting in deregulated expression [97,98] or formation of fusion proteins [99,100,101,102,103], the most common genetic alterations result in partial inactivation of one or several TFs [94,95,96]. Even though inherited point mutations in PAX5 have been reported to result in increased leukemia incidence [104], the reduced functional TF activity more commonly depends on the inactivation of the TF genes via somatic heterozygote mutations and deletions [94,95,96]. The importance of TF dose for normal blood cell development in mice is well established because heterozygote inactivation of the *Myb* [48], *Spi1* [105], *Bcl11a* [106], *Ikzf1* [107], or *Ebf1* [108] gene results in disturbances in B-cell differentiation. Despite the fact that the heterozygote inactivation of the *Pax5* gene in mice does not appear to result in any dramatic developmental block [109,110], it has been suggested to result in alterations of cellular metabolism that may promote transformation [111]. Furthermore, *PAX5* deletions are often found in combination with complex karyotypes and other genetic aberrations, including recurrent translocations like t(12;21)(p13;q22) (ETV6-RUNX1) or t(1;19)(q23;p13) (*TCF3*-*PBX1*) [94,96]. *PAX5* mutations are also found in combination with genetic alterations in other TFs such as *IKZF1* and *EBF1* [94,96], likely augmenting the effect of a reduced PAX5 dose. Interestingly, the phenotypic changes in B-cell development in mice carrying heterozygote inactivation of a TF gene are often exacerbated upon combined targeting of two TFs. Combined heterozygote inactivation of *Ebf1/Tcf3* [108], *Ebf1/Runx1* [112], or *Ebf1/Pax5* [113] results in more dramatic phenotypes than what is observed in the single mutants. This highlights the importance of functional and correctly balanced TF networks in B-cell development.

Even though leukemia is generally considered to be confined to one defined hematopoietic lineage, about 7% of patients display a more complex disease [114,115]. These malignancies are denoted as acute leukemia of ambiguous lineage (ALAL). ALAL can be manifested either as bi-lineal leukemia, involving several lineages, or bi-phenotypic disease, with expansion of cells displaying combined expression of normally lineage-restricted surface markers [114,115,116]. It has been reported that the level of PAX5 regulates the formation of bi-phenotypic leukemia [117] and that B-ALL cells carrying mutations in *Pax5* can be converted into other lineages with preserved malignant features [78,118]. Furthermore, in MYC-induced lymphoma, oscillations in EBF1 and PAX5 levels result in lineage plasticity [119]. Additionally, dramatic phenotypic changes with preserved cytogenetic features have occasionally been reported from patients experiencing relapse of disease [120], further challenging the idea that leukemia is restricted to a given lineage of cells. While this has been considered uncommon in clinical practice [114,115], novel treatment protocols may reveal a higher degree of complexity in B-ALL. Recently it was reported that 13 out of 20 patients relapsing after treatment of B-ALL with genetically manipulated T-lymphocytes (chimeric antigen receptor (CAR-T) cells) targeting CD19 developed CD19 negative leukemia even at the clonal level [121,122]. This makes lineage plasticity a central mechanism for resistance development upon targeted treatment of leukemia. Hence, disruptions in transcription factor networks in leukemia may impact not only the transformation process per se but may also underlie the development of resistance to lineage-targeted therapies.

## 5. Integration of External Signals and Transcription Factor Networks in Early B-Cell Development

Even though intrinsic cell events such as regulatory loops created by TF networks are of critical importance for B-cell differentiation, the normal development and expansion of progenitors depend on extracellular signals in the microenvironment. While some of these signals are shared with other hematopoietic progenitors, others are restricted to lymphoid progenitor compartments. Among the former are Kit ligand (Steel factor, Stem Cell Factor SCF), acting via the receptor tyrosine kinase cKIT [123]. This receptor is expressed in a variety of hematopoietic progenitor cells including the multipotent hematopoietic stem cells [124], myeloid progenitors [125,126] as well as the CLPs [7]. Subsequently, disruption of this signaling pathway results in defective formation of multiple hematopoietic lineages [127,128,129]. The expression of cKIT is rather restricted in B-lymphoid progenitors, but a substantial fraction of the pro-B cell compartment retains this surface receptor as well as an ability to respond to the cytokine [130]. CXCL12 is another broadly acting cytokine involved in the homing of cells to specific niches in the BM [131,132]. The protein acts via its surface receptor CXCR4 and both the ligand and the receptor are crucial for normal homing of hematopoietic progenitor cells [131,132], including B-cell progenitors [133]. The expression of CXCL12 is restricted to specific subpopulations of stroma cells in the BM [131], contributing to the ability of this chemokine to act as an organizer of the BM microenvironment.

B-cell development is also influenced by cytokines with more restricted activity. These include FLT3 ligand (FL) acting through the FLT3 receptor expressed in the earliest lineage-restricted progenitor cells. Both the LMPP [6] and the CLPs [134] express FLT3; however, upon progression of B-cell development, the expression is downregulated as a consequence of the *Flt3* gene being repressed by PAX5 [135]. Ectopic expression of the FLT3 ligand causes alterations in blood cell development [136] and disruption of this signaling pathway results in reductions in LMPPs and CLPs [137]. However, this occurs without dramatic changes to the peripheral CD19^+^ B-cell compartments [137]. The phenotype is exacerbated when combined with inactivation of Il7 signaling since this results in a complete block in BM B-cell development [138]. In line with the idea that the Il7R is expressed on CLPs with all lymphoid lineage potentials [7], lineage tracing analysis suggests that all B- and T-lymphoid cells as well as a substantial portion of the NK cells in the adult mouse have a history of Il7 expression [139]. The IL7 receptor is expressed on B-cell progenitors and a deficiency in either the receptor [140] or the ligand [141] results in a dramatic impairment in B-cell development already in the B-cell-restricted CD19^−^ compartments in the mouse BM [60,142]. The Il7 receptor α (IL7Rα) chain is also a component of the receptor for thymic stromal lymphopoietin (TSLP), a cytokine acting via the Il7Rα and a specific TSLP-receptor [143]. IL7 and TSLP appear to be functionally redundant since ectopic expression of the latter largely rescues B-cell development in IL7-deficient mice [144].

Many cytokines would appear to be permissive, stimulating proliferation and reducing apoptosis in the B-cell progenitors rather than driving development to a specific lineage in an instructive manner. However, while T-cell development can be largely rescued by overexpression of BCL2 in mice deficient in IL7 signaling, this does not fully rescue B-cell development [145,146]. This suggests partially distinct functions for IL-7 signaling in the formation of different lymphoid lineages. One potential explanation for this could be that STAT5 activation, resulting from IL7R signaling [147], induces *Ebf1* transcription, potentially driving the progenitor towards B-cell fate [148]. Furthermore, ectopic expression of EBF1 partially rescues B-cell development in mice lacking IL7 [148] or the BTB/POZ domain transcription factor ZBTB17 (MIZ1), crucial for functional IL7 signaling [149]. While this would suggest that IL7 has unique functions in the induction of the genetic program in early B-cell progenitors, the finding that ectopic expression of FL can rescue B-cell development independently of IL7 argues for a more permissive function [150]. Furthermore, the developmental block imposed by conditional deletion of STAT5, a key mediator of Il7 signaling [147], can be partially rescued by ectopic expression of anti-apoptotic proteins [151]. In all, it would appear that the IL7 signaling pathway has both permissive and instructive components, as supported by the finding that deletion of the pro-apoptotic protein BIM rescues survival but not differentiation of B-cell progenitors in Il7-deficient mice [152].

Despite the function of Il7 in human B-cell development being somewhat disputed [153], inactivating mutations in the common gamma chain results in severe combined immunodeficiency in humans [154] and activating mutations in the IL7 signaling pathway are commonly detected in human malignancies [155]. Interestingly, heterozygote deletion of *Pax5* or *Ebf1*, in combination with transgenic expression of a constitutive active STAT5, causes a synergistic increase in the formation of B-lineage leukemia [156]. Hence, the interplay between TF networks and extracellular signals is critical for normal B-cell development and disturbances may result in impaired immune response or lymphoid malignancies.

## 6. Concluding Remarks

While the detailed understanding of maturation pathways is often considered a subject mainly relevant to developmental biology, our increased understanding of molecular events involved in malignant transformation highlights the relevance of cell differentiation in malignant transformation. Understanding developmental trajectories can be important for diagnosis since, even though leukemia is caused by expansion of progenitor B-cells, the heterogeneous expression of surface IG suggests that leukemia can reside in both the pro- and pre-B cell compartments [114,115]. The use of more advanced FACS staining protocols may resolve an even higher heterogeneity and possibly better classify leukemia in both the CD19-positive and CD19-negative (ALAL) groups. Furthermore, it is becoming increasingly clear that the regulatory networks that drive normal development are targeted in the transformation process. This is knowledge that can be explored to identify novel diagnostic and therapeutic approaches. Additionally, the understanding of the molecular regulation of lineage stability can be used to predict the risk of relapse through lineage conversion in association with targeted therapies. Hence, it can be predicted that basic developmental biology will become of increasing importance for the improvement of modern cancer care in the near future.
